# Consequences of male partner engagement policies on HIV care-seeking in three African countries: Findings from the SHAPE UTT study

**DOI:** 10.1080/17441692.2020.1805788

**Published:** 2020-08-11

**Authors:** Albert Dube, Jenny Renju, Joyce Wamoyi, Farida Hassan, Janet Seeley, Rujeko Samanthia Chimukuche, John Songo, Thokozani Kalua, Amelia Crampin, Mosa Moshabela, Alison Wringe

**Affiliations:** a Malawi Epidemiology and Intervention Research Unit, Lilongwe, Malawi; b Department of population health, London School of Hygiene and Tropical Medicine, London, United Kingdom; c Department of Epidemiology and Biostatistics, Kilimanjaro Christian Medical University College, Moshi, Tanzania; d Mwanza Intervention Trials Unit, Mwanza, United Republic of Tanzania; e Ifakara Health Institute, Ifakara, United Republic of Tanzania; f Africa Health Research Institute, KwaZulu-Natal, South Africa; g University of Kwa-Zulu Natal, Durban, South Africa; h Department of HIV and AIDS, Malawi Ministry of Health, Lilongwe, Malawi

**Keywords:** HIV, sub-Saharan Africa, Option B+, partner engagement, Tanzania, Malawi and South Africa

## Abstract

We explored how strategies to promote male partner engagement influenced HIV care-seeking among men and women living with HIV. In-depth interviews were conducted with 25 health workers, 66 female service users and 10 male partners in Ifakara (Tanzania), Karonga (Malawi) and uMkhanyakude (South Africa) to elicit experiences of offering, providing or receiving HIV care in the context of antenatal care. Data were coded inductively and analysed thematically. Participants reported benefits of couple testing during antenatal care, including facilitated HIV status disclosure and mutual support for HIV care-seeking. However, unintended consequences included women attending without partners, being refused or delayed access to antenatal services. Some women were required to obtain letters from village leaders to justify the absence of their partners, again to delaying or disrupting care-seeking. When partners attended antenatal care, consultations were reportedly more likely to focus on HIV testing, and less on antenatal or neonatal care. Strategies to increase men’s attendance at HIV clinics with their partners can promote mutual support within couples for HIV care engagement, but may risk undermining engagement in pregnancy and HIV care for some women if over-stringently applied. Efforts are needed to address the underlying pervasive stigma associated with HIV care, both alone and as a couple.

## Introduction

In 2011, Option B+ for the prevention of mother-to-child transmission (PMTCT) of HIV was first coined and implemented in Malawi and involved the lifelong initiation of antiretroviral therapy (ART) to all pregnant and post-partum women regardless of their immune status (The Ministry of Health Malawi, 2013). The same strategy was then recommended by the World Health Organisation in 2013 and rapidly adopted across sub-Saharan Africa (World Health Organisation (WHO), 2013). The expansion of Option B+ programmes for the prevention of mother-to-child transmission (PMTCT) of HIV in sub-Saharan Africa, has led to increased HIV testing and treatment coverage among women of a reproductive age. By 2017, it was estimated that 76% of women living with HIV in the region knew their HIV status, an increase from 72% in 2015. However, for various reasons HIV diagnosis rates are lower and subsequent HIV related mortality is higher among men (Bhatta et al., 2013; Poka-Mayap et al., 2013). In 2016 deaths from AIDS-related illnesses were 27% lower among women and girls than they were among men and boys (UNAIDS, 2017).

Despite improvements in PMTCT uptake and outcomes over the past decade, studies have shown that numerous barriers persist at the individual, community and health systems levels, impacting service uptake (Gourlay et al., 2014; Knettel et al., 2018; Whembolua et al., 2019). Factors relating to decision-making within families and issues within relationships also frequently emerge as being key drivers of women’s engagement with PMTCT services. Male partner participation in antenatal care (ANC) services has been proposed to improve PMTCT uptake, as well as to improve men’s diagnosis and treatment rates, and is recommended by the World Health Organization (WHO) (Aluisio et al., 2016; Osoti, 2014; van den Berg et al., 2015; World Health Organisation (WHO), 2012). Studies in sub-Saharan Africa have demonstrated that male participation in antenatal care (ANC) can increase HIV testing and treatment uptake among pregnant women, improve women’s retention in care and improve HIV-free survival among infants (Audet, Blevins, et al., 2016; Takah et al., 2018; Vrazo et al., 2018; Wesevich et al., 2017). Male partner involvement has also been linked to the readiness of pregnant women accepting antiretroviral treatment (ART) on the same day as receiving an HIV diagnosis at ANC (Wamoyi et al., 2017). In Malawi, couple testing in the context of PMTCT programmes has also been shown to increase social support for women, including those with recent HIV diagnoses, (Bhushan et al., 2019) and to increase condom use in couples with an HIV-infected pregnant woman (Rosenberg, Graybill, et al., 2017). However, other studies have reported that male attendance at ANC clinics may contribute to stigmatisation of women if it is interpreted by people in the community as a sign that the woman is HIV-positive (Audet, Chire, et al., 2016).

Some studies from Eastern and Southern Africa have explored the role of male engagement in ANC on outcomes for the couple as a unit, finding that men and women reported that partner involvement at ANC strengthened relationships and promoted mutual support for HIV-related behaviours (Rosenberg, Gross, et al., 2017; Takah et al., 2017; Wamoyi et al., 2017). However far less research has investigated how attending ANC appointments together impacts on the experiences of women and men, including in testing or diagnosis rates (Theuring et al., 2009). Factors influencing men’s involvement in ANC or PMTCT services often relate to underlying social and gender norms that define relationship dynamics, the role of men in pregnancy and childcare, and men’s care-seeking behaviour more generally (Wamoyi et al., 2017). Many sub-Saharan countries (including Malawi, Tanzania and South Africa) have adopted policies to promote male involvement in sexual and reproductive health and PMTCT services (Ministry of Health Community Development Gender Elderly and Children, The United Republic of Tanzania, 2017; Ministry of Health and Social Welfare, The United Republic of Tanzania, 2013; The Ministry of Health Malawi, 2016). These policies have resulted into strategies such the promotion of couples‘ attendance at antenatal care services and couples counselling and testing for HIV and subsequent services.

Despite the growing body of evidence supporting male involvement in PMTCT programmes, and emerging policy guidance on HIV care-seeking for couples during pregnancy and beyond, few studies have explored the consequences of male engagement strategies for men and women as they engage with pregnancy and HIV services together. This is particularly pertinent now in the era of universal test and treat, unlike under previous treatment strategies, where both pregnant and non-pregnant women and men who tests HIV positive are immediately eligible for ART initiation. In this paper, we draw on qualitative data from a larger study on the health systems impacts of PMTCT to explore how the implementation of male engagement policies impact women and men’s experiences of health-seeking in rural Malawi, Tanzania and South Africa.

## Methods

### Study sites

In this analysis, we draw on data from the study for “Strengthening Health Systems for the Application of Policy to Enable Universal Test and Treat” (SHAPE UTT) which aimed to investigate the health systems impacts of delivering Test and Treat within three rural settings: Ifakara in southern Tanzania, Karonga in northern Malawi, and uMkhanyakude in north-eastern South Africa (Table 1).

### Sampling and recruitment

From October 2017 to December 2018, we conducted in-depth interviews with health service providers, female health service users and their male partners in Ifakara, Karonga and uMkhanyakude.

We purposively sampled service providers from three health facilities in each site to ensure a range of roles and cadres. In each facility we requested to speak to the providers who were primarily responsible for ANC care and specifically HIV testing within ANC visits. So as to not interfere with work, we invited health workers to participate in the study during quiet periods at work, or before the start of the working day. We sampled female service users from all participating health facilities to include ANC users, women living with HIV and enrolled in the Option B+ PMTCT program, HIV clinic users and women who were lost to follow up (LTFU) from Option B+ programmes. To do this we developed a sampling frame from the facility survey registers, recruitment was facilitated by expert clients in Karonga and health service providers in uMkhanyakude and Ifakara for those attending consultations, and by expert clients (Karonga) and patient tracers (uMkhanyakude and Ifakara) for those who were lost to follow up. In uMkhanyakude and Ifakara we contacted male partners of the female service users who participated in the study and who granted us permission to contact their partner. A total of 101 participants were recruited. Sixty-six were female service users, of whom 21 were enrolled in Option B+, 17 were lost to follow up from Option B+, 15 were ANC service users and 17 were enrolled in routine HIV care. Ten male partners and 25 health workers were also interviewed. The majority of health workers were nurses. (Table 2)

### Data generation

All the interviews were semi-structured and guided by a topic guide complete with prompts. The topics included in the semi-structured interviews with health workers included processes of providing care to women and their partners, with additional probes to explore strategies they used to promote couples attendance, what they would do if the couple did not attend together and what they saw as the facilitators, barriers and subsequent effects of couples attending ANC and HIV testing together. Our semi-structured interviews with women and their partners covered their relationships and their experiences of using ANC and HIV services. Additional probes explored the experiences of the individuals with couple’s engagement in care, and the way they were encouraged to seek care with their partner, and how they felt about these strategies and their views on the subsequent effects of these strategies.

All interviews were conducted by trained, non-clinical social scientists who had extensive field worker experience and took place in a private setting within the clinic environment, interviewees were asked if they were comfortable in the setting prior to starting the interview. Interviews were conducted in English with health workers and in the local vernacular with service users and male partners, and lasted between 60 and 90 min. No information from the discussions with female service users was disclosed during interviews with their partners, which were undertaken by a different field worker.

Following each interview, fieldworkers prepared detailed notes and underwent a debriefing with the study coordinators, providing an opportunity for on-the-job training, reflection on emerging topics, and an assessment of whether saturation was being reached.

### Data analysis

All interviews were audio-recorded, transcribed and translated into English. The study coordinator in each setting read the transcripts to familiarise themselves with the data. The study coordinators applied country-specific broad coding in each setting aided by Nvivo 11 (Tanzania and Malawi) and manually in word and excel (South Africa), which related to the broader SHAPE study. One of the broad themes related to male involvement in ANC. This broad coding theme was extracted from each country database and exported to the lead author. Coding was then undertaken inductively (Mason, 2002) and iteratively to explore how male engagement affected both male and female care engagement. Emerging findings were then clustered to produce themes. Themes were regularly compared and discussed by the study coordinators in each site to reflect on emerging findings in relation to contextual differences. Interviews between female service users and male partners were not analysed dyadically for this sub-study.

### Ethics

Ethical approval was obtained from the institutional review boards of the Ifakara Health Institute (14-2017), the National Institute for Medical Research for Tanzania (2579), the National Health Science Research Committee for Malawi (1861), the University of KwaZulu-Natal Biomedical Research Committee in South Africa (BE400/14), and the London School of Hygiene and Tropical Medicine (13536-1). Information sheets were provided and read to each participant, questions were welcomed and answered and then all participants provided written informed consent to both the interview and to the use of a digital recorder. No financial remuneration was provided however refreshments were offered in the form of a drink and snack.

## Results

Three themes emerged through the analysis: strategies employed by health workers to promote male attendance with their partner at the ANC visits, the perceived value of male involvement in promoting HIV care engagement, and consequences of male involvement on men and women’s HIV care-seeking.

### Strategies adopted to promote male attendance

Service providers described various strategies they implement to promote male partner attendance at ANC and PMTCT. Some providers, in Tanzania, reported to schedule special appointments for newly presenting couples at ANC services in order to accommodate the perceived needs of the male partner. In both the Malawian and Tanzanian sites, it was often suggested by providers that men should be able to access the service with ‘comfort’, and should not be kept waiting long before being served. When asked for the reasons for prioritising men in this way, some service providers suggested that if men were to spend the whole day at the facility, this would discourage other men from attending with their partners:

… we make sure we have scheduled days (Wednesday and Friday) for new patients and we make sure we don’t have appointments with those other regular patients so that the new patients can feel comfortable on their first day, we also do not keep the male partners waiting for services for a long time, if we do they will tell their friends (health worker −Ifakara, Tanzania)

During ANC women have to undergo multiple tests, some health workers in Ifakara, Tanzania, described how some tests were conducted on different days to ensure that the male partners would not have to wait for long periods of time. In some facilities in Ifakara, a women’s first ANC visit would often be conducted over two days, one day for all the required tests, which can be done in the absence of the male partner and the ‘partner may join their spouses on the second day’. In Karonga and uMkhanyakude, however, service providers described how emphasis was placed on conducting all services together as a couple and on the same day.

### Perceived value of male involvement in PMTCT

Health workers and female services users, regardless of whether attending PMTCT or ANC services, reported various benefits of couples attending HIV testing together. Many female service users in Karonga and Ifakara highlighted that when women attended with their partners, they were often served more quickly than those who came by themselves. The women who came with their partners, appreciated the reduced waiting times. Some health workers in Malawi mentioned that when men attended clinics, it became easier to mutually disclose their HIV status and initiate ART treatment and supporting each other’s engagement in care including collecting drugs when one is ill or busy. Additionally, some providers and female service users felt that the inclusion of partners provided benefits for the unborn child, both in terms of preparing for the birth and in protection from HIV.

‘We normally provide services for a woman and husband together on their first visit [so that] they both get the service together because our purpose is to save the baby that is in the mother’s womb … ’ (Health worker, Ifakara, Tanzania).

Some health workers reported that men received health advice (including HIV results) better when told by the Health workers themselves rather than their spouses.

‘sometimes nurses or doctors will tell women that the time has come to stop some domestic chores because of their condition and sometimes when men hear it from their spouses they don’t believe them, they feel their wife is being lazy, so when they are together they can also listen to the advice given to them as a family’ (Health worker, Karonga, Malawi).

It was also reported by some women that attending with their husbands made it easier for the husbands to buy necessities such as a piece of cloth that women wear around the waist, also used for carrying babies, a basin, a baby blanket, baby socks, a hat and napkins, that are recommended for the unborn child and the mother during the time of delivering the child.

‘When I go alone to ANC, I am told things. When I come home and tell my husband about what the health workers have advised us to purchase to prepare for the child, he will not understand. Instead he will argue and say it’s my personal demand. But the good thing about attending ANC together is that both of us hear information about the required materials together’ (routine ART user-Karonga, Malawi)

In all sites, participants reported that for some, couple’s involvement in PMTCT at ANC could lead to a greater level of mutual support between partners who attended services together.

She makes sure that we eat first, before taking the medication. If there is nothing, I try my best to find small jobs so that we can get food. We put things together since our lives are at stake” (Male partner, uMkhanyakude, South Africa).

Some women who sought services together with their partners and were found to be HIV discordant, described instances where their partners were more understanding and accommodating of the HIV status of their partners regardless of their own HIV status.

… I should not hide. At first I refused and I said: “I accept whatever may come to me“ Later he started encouraging me that I should get drugs. He said I should not think that he will leave me because I tested HIV positive, and that I have to get drugs … (woman, routine ART user, Karonga, Malawi)

### Unintended consequences of strategies to promote male attendance

We found several unintended consequences of the strategies adopted by service providers to promote couple engagement in care emerged. Whilst the prioritisation of women who attended ANC with their partners was valued, the flip side was that women who did not attend with partners were disadvantaged. Health workers and female participants from Karonga and Ifakara described experiences of women who were unable to attend with their partner being seen at the end of the clinic, pushed on to another day or just denied services altogether. Many women who attended without partners reported having longer waiting times and sometimes felt resentful that they waited longest to be served, even if they were the first to arrive:

But those who came with their husbands, even if they come late, they were the first ones assisted and they went home earlier than us … ’ (Woman, lost-to follow-up, Karonga, Malawi).

Some women and service providers reported delays or refusals in offering ANC attendees an HIV test, or in starting ART treatment among women with a positive test result if their male partners were not present for the consultation. Additionally, health workers reported that in some instances male partners lack of readiness to test and/or start treatment could negatively impact on the female partners’ care engagement, despite the health workers best efforts to promote engagement.

In some instances, women were expected to wait for their partners to find time to visit the facility together. Subsequently, for women who were delayed or refused appointments until they came with their partner, delays were reported in both HIV diagnosis (delayed testing) and treatment initiation.

We had this one patient she said that she is waiting for her partner who has travelled to come back so they could come together and start treatment’ (Health worker, Ifakara, Tanzania)

In Ifakara and Karonga, some women reported needing to present a letter from their village leader to justify the absence of their partner from ANC consultations, in order to remain eligible for the services

For those who have carried letters from village head, they raise up their hands. For those who have come without their partners or letters from village heads, they are told to go home. They are advised to attend antenatal clinic if they come with their partners or a witness letter from village head (routine ART user, Karonga, Malawi)

Some health workers reported that they had witnessed conflicts among couples if they received discordant results. Some Health workers found it challenging to properly manage the conflicts that erupted after receiving results. Furthermore, some couples found it difficult to understand discordant results when they have been together a long time. For some who were found to be HIV discordant, health workers reported seeing couples separating and eventually divorcing.

‘The difficulty comes as they are a couple, they have been living together for maybe two to three years and they trust each other, and all of a sudden you tell them that one is infected (positive) and other one is not, it’s quite difficult to digest the situation, and it is difficult to counsel them on how to accept the situation and how to live knowing that your partner is infected, sometimes because of this, they get separated’ (Health worker, Ifakara, Tanzania).

In uMkhanyakude, some partners described instances where they had shared the ART medication of their partner when theirs had run out and they were unable to get the refill or the partner was too busy to go and collect it on their behalf.

Yes, because even when I am busy and she is also preoccupied, I sometimes use her medication and drink it up until I find time to go and collect mine at the clinic (Male-partner, uMkhanyakude, South Africa).

Finally, reports from all sites suggested that when partners attended ANC consultations the content was primarily focused on HIV testing. The process involved couples pre and post-test counselling with particular care needed should the couple receive a discordant result. It was clear that the focus on the HIV test lessened the focus on ANC, meaning that topics pertaining to care of the new-born, breast feeding, maternal health were left to be covered in subsequent visits.

## Discussion

**Figure 1:**
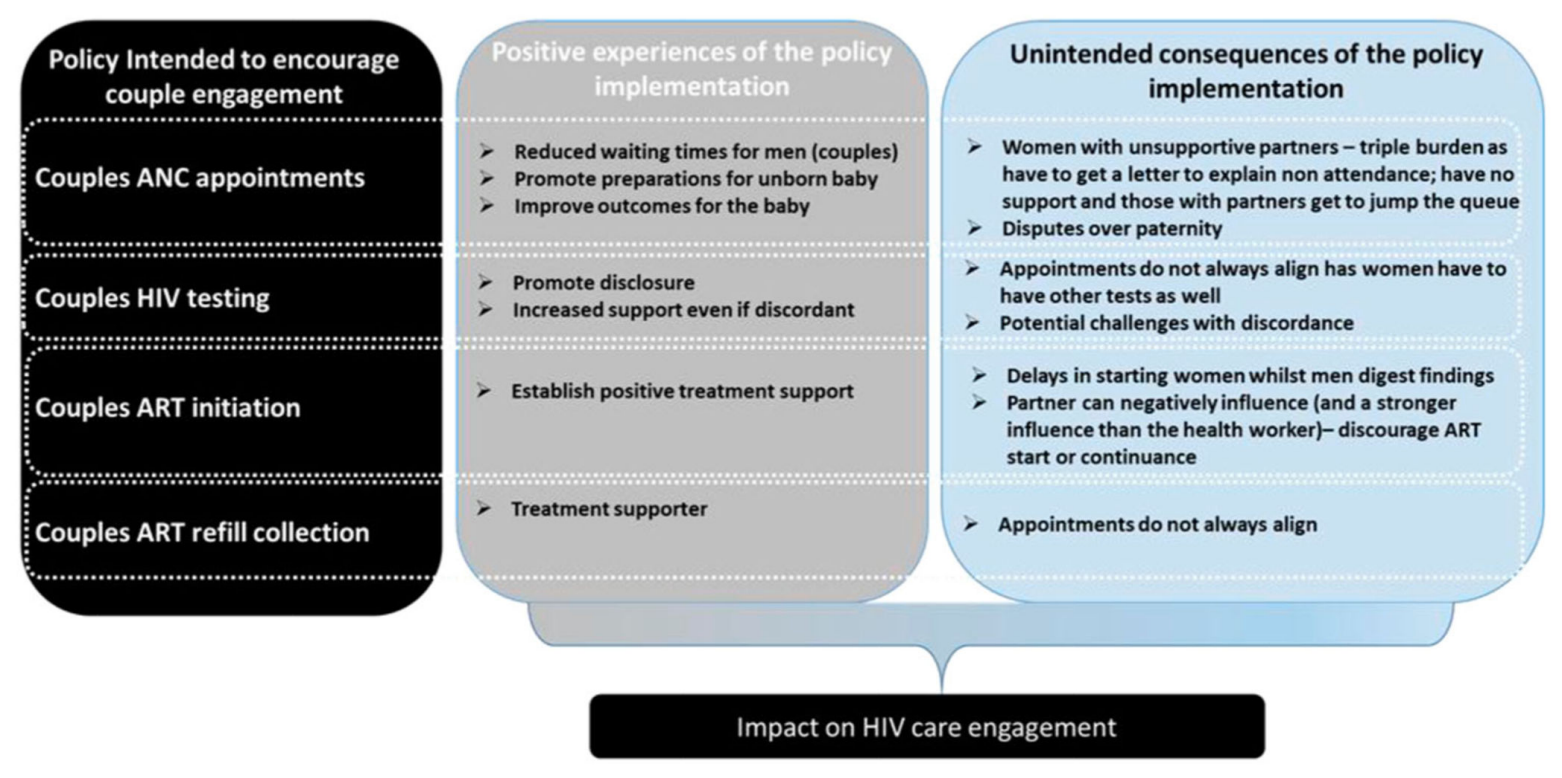
Schematic to illustrate how the different positive experiences and unintended consequents of couple engagement policies at different points on the HIV care cascade can affect HIV care engagement.

The facilities in this study adopted different strategies to promote the involvement of men in PMTCT within ANC which have manifested in various positive and negative outcomes. These outcomes occur at different stages of the HIV cascade of care from the initial HIV test at the first ANC appointment through ART initiation and to continued retention in care in all the three study settings (Figure 1). Our study suggests that some of the strategies adopted to encourage men to attend the ANC and HIV services sometimes enhanced disclosure and led to increased support between the couples in taking their HIV treatment. On the other hand, we found that the well intentioned enforcement of strategies to achieve male engagement can disadvantage some women.

Many studies have demonstrated benefits of male engagement in reproductive health services. Studies in sub-Saharan Africa showed that male participation in ANC can increase HIV testing and treatment uptake among pregnant women, improve women’s retention in Option B+ programmes and improve HIV-free survival among infants (Audet, Blevins, et al., 2016; Takah et al., 2018; Vrazo et al., 2018; Wesevich et al., 2017). Specifically, our findings also highlight positive impacts of facility level strategies employed by health workers such as: reduced waiting time for men, promotion of preparedness for the unborn child and increased partner support in discordant couples. Like other studies in the region, we heard of experiences of how a joint HIV diagnosis enabled couples to support each other in their HIV treatment. Evidence also suggests that a well-supported entry into HIV care and treatment at ANC could actual strengthen relationships (Rosenberg, Graybill, et al., 2017; Wamoyi et al., 2017).

However, on the contrary, some of the strategies used by health workers to encourage male involvement had negative consequences and thus discouraged women from seeking services. Studies have reported that many women and men face barriers when accessing PMTCT programmes (Audet, Blevins, et al., 2016; Audet, Chire, et al., 2016; Clouse et al., 2014; Gourlay et al., 2013; Morfaw et al., 2013). Our study revealed a number of negative consequences of facility level strategies. Specifically, participants (both women and health workers) reported that should they come without a partner they would have to take time to obtain letters from the local authorities prior to receiving services. In this Tanzanian setting, some of the women who came with a partner had to avail an additional day to ensure that their partner ‘wasted less time’ waiting for the other ANC testing services. This suggests men’s time holds a greater value than that of women. Women with unsupportive partners experienced a huge burden of having to get a letter from village heads to explain non-attendance of their male partners while those with their partners were fast-tracked for services, subsequently further disadvantaging those that came early but without their partners. Women’s individual circumstances need to be taken into account to ensure that that those in most need are not further disadvantaged, nor is their subsequent HIV and ANC care engagement affected.

HIV remains a very stigmatised condition in sub-Saharan Africa, creating a great deal of apprehension surrounding testing, disclosure, and care engagement. Stigma and its manifestations within HIV care uptake and retention have been well document (Bonnington et al., 2017; Cichowitz et al., 2019; Watt et al., 2019). These and other studies repeatedly show how stigma affects how women think about themselves personally (internalised stigma) leading to fears as to how they would be able to live positively within their communities (anticipated and enacted stigma). In the context of such tensions, it is critical to minimise barriers to care, build positive rapport, and positive counsel/engage people who test positive. Careful consideration needs to be given to how attempts at inclusion of male partners could be further sparking stigma and/or negatively influencing factors that should be facilitating care engagement.

This qualitative study provided an in-depth exploration of couples’ HIV care engagement from the perspective of health workers, women living with HIV, women attending ANC and male partners in three different countries with different policy contexts. However, the findings should be interpreted with the following limitations in mind: firstly, our study took place in a small sample of facilities in rural settings and were not intended to be representative of urban settings, thereby affecting the generalisability of our findings. Secondly participant’s responses may be influenced by social desirability bias, however our triangulation of data from three different participant categories (women, men and service providers) strengthened our ability to explore thematic areas from different perspectives. Thirdly we were only able to recruit a small number of male participants into the study and the individuals within the couples were not interviewed together, potentially further perpetuating a social desirability bias. In future studies, we would recommend adopting a more ethnographic approach involving a larger sample of linked couples to allow for a greater depth of opportunities to unpick the complexities of relationships and care engagement in the context of ANC.

## Conclusion

Facility level strategies to increase men’s attendance at HIV clinics with their partners may promote their earlier diagnosis and lead to mutual support within couples for treatment engagement. However, if they are over-stringently applied, they may undermine engagement in pregnancy and HIV care for some women. Flexible and tailored approaches are needed to ensure that some socially vulnerable women are not further disadvantaged when seeking antenatal and HIV care.

## Figures and Tables

**Table 1:** Study Setting Characteristics.

Country	Tanzania		Malawi		South Africa	
Year of country adoption of Option B+	2013		2011		2015	
Year of country adoption of UTT	2016		2016		2016	
Characteristics of Health and Demographic Surveillance Sites (HDSS)						
Location of HDSS	Ifakara		Karonga		uMkhanyakude	
Year HDSS established	1996		2002		2000	
Population of HDSS	135,000		40,000		90,000	
HIV prevalence in HDSS	7%		9.6%		33%	
Total number of health facilities in HDSS	11		5		17	
Facility types	*N*	%	*N*	%	*N*	%
Dispensary	2	18	0	0	0	0
Small clinic	0	0	0	0	10	59
Large clinic/small health center	4	36	3	60	7	41
Large health center /sub-district hospital	3	27	2	40	0	0
District hospital	1	9	0	0	0	0
Referral hospital	1	9	0	0	0	0
Government	9	82	3	27	17	100
Faith-based organisation	1	9	2	18	0	0
Private-for-profit	1	9	0	0	0	0

**Table 2:** Study participants.

Country HDSS site	Tanzania Ifakara	Malawi Karonga	South Africa uMkhanyakude	Total
**In-depth Interviews**				
*Service users*				
**PMTCT B+ service users:** HIV positive pregnant and postpartum women	8	5	8	21
**ANC users:** HIV negative pregnant women	4	5	6	15
**HIV routine service users:** HIV positive postpartum women graduated from PMTCT	4	7	6	17
**PMTCT lost to follow up:** HIV positive pregnant and postpartum women disengage in treatment	2	5	6	13
**Male partners**	5		5	10
*Health workers*				
HTS counsellor		1		1
Nurse-midwife technician	4	3		7
HIV diagnostic assistant		1		1
Expert client		2		2
Professional nurse	1		5	6
Enrolled nurse		5	1	6
Nurse Assistant	2			2
**TOTAL**	**30**	**34**	**37**	**101**

## Data Availability

Data will be available for the period of 5 years from the end date of the study upon request to the study PI: Alison.Wringe@lshtm.ac.uk

## References

[R1] Aluisio AR, Bosire R, Bourke B, Gatuguta A, Kiarie JN, Nduati R, Farquhar C (2016). Male partner participation in antenatal clinic services is associated with improved HIV-free survival among infants in Nairobi, Kenya: A prospective cohort study. Journal of Acquired Immune Deficiency Syndromes.

[R2] Audet CM, Blevins M, Chire YM, Aliyu MH, Vaz LME, Antonio E, Vermund SH (2016). Engagement of Men in antenatal care services: Increased HIV testing and treatment uptake in a community Participatory Action program in Mozambique. AIDS and Behavior.

[R3] Audet CM, Chire YM, Vaz LME, Bechtel R, Carlson-Bremer D, Wester CW, Gonzaléz-Calvo L (2016). Barriers to male involvement in antenatal care in rural Mozambique. Qualitative Health Research.

[R4] Bhatta L, Klouman E, Deuba K, Shrestha R, Karki DK, Ekstrom AM, Ahmed LA (2013). Survival on antiretroviral treatment among adult HIV-infected patients in Nepal: A retrospective cohort study in far-western region, 2006-2011. BMC Infectious Diseases.

[R5] Bhushan NL, Golin CE, McGrath N, Maman S, Tsidya M, Chimndozi L, Rosenberg NE (2019). The impact of HIV couple testing and counseling on social support among pregnant women and their partners in Lilongwe, Malawi: An observational study. AIDS Care.

[R6] Bonnington O, Wamoyi J, Ddaaki W, Bukenya D, Ondenge K, Skovdal M, Wringe A (2017). Changing forms of HIV-related stigma along the HIV care and treatment continuum in sub-Saharan Africa: A temporal analysis. Sexually Transmitted Infections.

[R7] Cichowitz C, Mazuguni F, Minja L, Njau P, Antelman G, Ngocho J, Mmbaga BT (2019). Vulnerable at each Step in the PMTCT care cascade: High Loss to follow Up during pregnancy and the postpartum period in Tanzania. AIDS and Behavior.

[R8] Clouse K, Schwartz S, Van Rie A, Bassett J, Yende N, Pettifor A (2014). What they wanted was to give birth; nothing else: Barriers to retention in option B+ HIV care among postpartum women in South Africa. Journal of Acquired Immune Deficiency Syndromes.

[R9] Gourlay A, Birdthistle I, Mburu G, Iorpenda K, Wringe A (2013). Barriers and facilitating factors to the uptake of antiretroviral drugs for prevention of mother-to-child transmission of HIV in sub-Saharan Africa: A systematic review. Journal of The international Aids Society.

[R10] Gourlay A, Wringe A, Birdthistle I, Mshana G, Michael D, Urassa M (2014). “It Is Like that, We Didn't understand each other”: Exploring the Influence of patient-Provider Interactions on prevention of mother-To-child transmission of HIV service Use in rural Tanzania. PLOS ONE.

[R11] Knettel BA, Minja L, Chumba LN, Oshosen M, Cichowitz C, Mmbaga BT, Watt MH (2018). Serostatus disclosure among a cohort of HIV-infected pregnant women enrolled in HIV care in Moshi, Tanzania: A mixed-methods study. SSM – Population Health.

[R12] Mason J (2002). Qualitative Researching 2nd Edition Qualitative Research Book.

[R13] Ministry of Health and Social Welfare, & The United Republic of Tanzania (2013). National Guidelines for Comprehensive Care Services for Prevention of Mother-to-Child Transmission of HIV and Keeping Mothers Alive.

[R14] Ministry of Health Community Development Gender Elderly and Children, The United Republic of Tanzania (2017). The National Guildelines for the management of HIV and AIDS.

[R15] The Ministry of Health Malawi (2013). Consolidated Guidelines for the Use of ART for Treating and Preventing HIV Infection.

[R16] The Ministry of Health Malawi (2016). HIV testing Services Guidelines.

[R17] Morfaw F, Mbuagbaw L, Thabane L, Rodrigues C, Wunderlich AP, Nana P, Kunda J (2013). Male involvement in prevention programs of mother to child transmission of HIV: A systematic review to identify barriers and facilitators. Systematic Reviews.

[R18] Osoti A (2014). Role of male partners in the prevention of mother-to-child HIV transmission. Research and Reports in Neonatology.

[R19] Poka-Mayap V, Pefura-Yone EW, Kengne AP, Kuaban C (2013). Mortality and its determinants among patients infected with HIV-1 on antiretroviral therapy in a referral centre in Yaounde, Cameroon: A retrospective cohort study. BMJ Open.

[R20] Rosenberg NE, Graybill LA, Wesevich A, McGrath N, Golin CE, Maman S, Miller WC (2017). The impact of couple HIV testing and Counseling on Consistent condom Use among pregnant women and their male partners: An Observational study. Journal of Acquired Immune Deficiency Syndromes.

[R21] Rosenberg NE, Gross R, Mtande T, Maman S, Golin CE, Saidi F, Miller WC (2017). “We have heard it together”: a qualitative analysis of couple HIV testing and counselling recruitment in Malawi’s Option B+ programme. African Journal of Aids Research.

[R22] Takah NF, Atem JA, Aminde LN, Malisheni M, Murewenhema G (2018). Male partner involvement in increasing the uptake of infant antiretroviral prophylaxis/treatment in sub Saharan Africa: A systematic review and meta-analysis. BMC Public Health.

[R23] Takah NF, Kennedy ITR, Johnman C (2017). The impact of approaches in improving male partner involvement in the prevention of mother-to-child transmission of HIV on the uptake of maternal antiretroviral therapy among HIV-seropositive pregnant women in sub-Saharan Africa: A systematic review and m. BMJ Open.

[R24] Theuring S, Mbezi P, Luvanda H, Jordan-Harder B, Kunz A, Harms G (2009). Male involvement in PMTCT services in Mbeya region, Tanzania. AIDS and Behavior.

[R25] UNAIDS (2017). Ending AIDS, Progress towards the 90-90-90 Targets.

[R26] van den Berg W, Brittain K, Mercer G, Peacock D, Stinson K, Janson H, Dubula V (2015). Improving men’s participation in Preventing mother-to-child transmission of HIV as a maternal, neonatal, and child health Priority in South Africa. PLoS Medicine.

[R27] Vrazo AC, Firth J, Amzel A, Sedillo R, Ryan J, Phelps BR (2018). Interventions to significantly improve service uptake and retention of HIV-positive pregnant women and HIV-exposed infants along the prevention of mother-to-child transmission continuum of care: Systematic review. Tropical Medicine & International Health.

[R28] Wamoyi J, Renju J, Moshabela M, McLean E, Nyato D, Mbata D, Wringe A (2017). Understanding the relationship between couple dynamics and engagement with HIV care services: Insights from a qualitative study in eastern and Southern Africa. Sexually Transmitted Infections.

[R29] Watt MH, Knippler ET, Minja L, Kisigo G, Knettel BA, Ngocho JS, Mmbaga BT (2019). A counseling intervention to address HIV stigma at entry into antenatal care in Tanzania (Maisha): study protocol for a pilot randomized controlled trial. Trials.

[R30] Wesevich A, Mtande T, Saidi F, Cromwell E, Tweya H, Hosseinipour MC, Rosenberg NE (2017). Role of male partner involvement in ART retention and adherence in Malawi’s Option B+ program. AIDS Care.

[R31] Whembolua G-LS, Muvuka B, Tshiswaka DI, Conserve DF (2019). Socio-Structural factors influencing the prevention of mother-to-child transmission of HIV in the Democratic Republic of the Congo: A Systematic review. Maternal and Child Health Journal.

[R32] World Health Organisation (WHO) (2012). Programmatic Update Use of Antiretroviral Drugs for Treating Pregnant Women and Preventing Hiv Infection in Infants Executive Summary.

[R33] World Health Organisation (WHO) (2013). Consolidated ARV guidelines.

